# Chemistry and the War

**Published:** 1918-11

**Authors:** 


### Chemistry and the War.

Chemistry is playing no little part in winning the war, and the war in return is making valuable contributions to chemistry. When the war is over this country will no longer have to turn to Germany for most of its chemicals, as it once did. Our chemists are proving their ability to cope with the world and they have made remarkable advances in the science of chemistry in the few years the world has been at war.

The American chemist's place in industry, a stocktaking of his past accomplishments and a caretaking for the permanency of his additions to the national wealth, whereby economic independence may be assured, were phrases broadly characterizing the recent fourth National Chemical exposition. The exposition was the largest and most significant of its kind ever held in America. It illustrated the contributions of the chemist toward the winning of the war, and the Government considered it of such importance that it was allowed to use the palace, although taken over for war purposes.

The hundreds of exhibits were of interest to the layman as well as the experts. There were motion pictures, displays illustrating the strides made by the dye industry, technical sessions of chemical societies, attended by hundreds of chemists from all parts of the country; machinery, and other features showing chemical progress.

Dr. Charles H. Herty, chairman of the advisory committee, points out that during the first eight months of 1918 the sum of $59,164,000 was added, making the aggregate authorized capital invested since Aug. 1, 1914, about $386,967,000, excluding the investments made by the Government in chemical plants doing war work. The total production of these plants makes the American Government the largest manufacturer of chemicals.

"The present status of the American chemical industry," says Dr. Herty, "and its prospects for the future, must prove gratifying to all good citizens, but these prospects can never be fully realized unless the work of the chemist is supported by sound and loyal public opinion, which, in turn, will eventually manifest itself in the form of a thoroughly sympathetic attitude on the part of official representatives of that public opinion.''

